# Social anxiety disorder and the psychobiology of self-consciousness

**DOI:** 10.3389/fnhum.2015.00489

**Published:** 2015-09-23

**Authors:** Dan J. Stein

**Affiliations:** Department of Psychiatry and MRC Unit on Anxiety and Stress Disorders, University of Cape TownCape Town, South Africa

**Keywords:** social anxiety disorder, psychobiology, self-consciousness, insula, temperoparietal

## Abstract

Individuals with social anxiety disorder (SAD) are characterized by fear or anxiety about social situations, but also by important alterations in self-referential processing. Given advances in our understanding of the neurocircuitry and neurochemistry of SAD, the question arises of the relationship between this research and an emergent literature on the psychobiology of self and self-consciousness. A number of investigations of SAD have highlighted altered activity in the medial prefrontal cortex (mPFC; involved in self-representation), insula (involved in interoceptive processing), and other structures that play a role in bodily self-consciousness, as well as the potential value of interventions such as selective serotonin reuptake inhibitors (SSRI) and self-focused reappraisal in normalizing such changes. Future studies to more closely investigate associations between psychobiological alterations and changes in self-related processing in SAD, may be useful in shedding additional light on both SAD and self-consciousness.

Social anxiety disorder (SAD or social phobia) is characterized in DSM-5 in terms of fear or anxiety about social situations in which the individual is exposed to possible scrutiny by others (American Psychiatric Association, 2013). Patients with SAD may have important alterations in self-referential processing (Gaebler et al., 2014; Jazaieri et al., 2015), and self-consciousness in SAD may be associated with increased severity of social anxiety (Hope and Heimberg, 1988). Given advances in our understanding of the neurocircuitry and neurochemistry of SAD (Hattingh et al., 2012; Fox and Kalin, 2014), the question arises of the relationship between this research and an emergent literature on the psychobiology of self and self-consciousness (Northoff et al., 2011)? Here I briefly address this issue.

## Sad and Self-Consciousness

To begin with, it is important to establish the nature of self-consciousness in SAD. A distinction has been drawn between public self-consciousness, or awareness of the public aspects of the self, and private self-consciousness, or awareness of one’s thoughts and feelings (Hope and Heimberg, 1988). SAD patients with high public self-consciousness were found to have more social anxiety, while those with high private self-consciousness where found to report more extensively and more accurately on their internal states (Hope and Heimberg, 1988). Still, the literature in this area is surprisingly sparse, and a good deal remains to be learned about the predictors and associations of self-consciousness and related phenomena in SAD (Schlenker and Leary, 1982; Panayiotou et al., 2014).

A construct that may be partially related to self-consciousness is that of self-focused attention. This has been defined in terms of awareness of self-referent, internally generated information, and may include awareness of thoughts and feelings, as well as of body state information (Ingram, 1990). Ingram suggested that excessive self-focused attention or self-absorption is involved in pathogenesis of a number of mental disorders, but with aspects of altered attention differentiating various conditions. Subsequent reviews have emphasized that self-focused attention is associated with increases in social anxiety, poor social performance, and negative self-judgments and attributions, with reduction following successful treatment of SAD (Spurr and Stopa, 2002), and have argued that attentional strategies should be targeted during SAD treatment (Bögels and Mansell, 2004).

Certainly, an influential model of cognitive-behavioral therapy (CBT) for SAD is based on the notion that self-focused attention is an important maintaining factor in SAD, increasing access to negative thoughts and self-imagery during social situations (Clark and Wells, 1995). That said, there appear to be a range of information-processing biases in SAD (Hirsch and Clark, 2004), and another influential CBT model emphasizes that in SAD there is attention to both internal cues and external stimuli indicative of negative evaluation (Schultz and Heimberg, 2008). Trials of attention bias modification (ABM) in SAD are not persuasive, although this perhaps reflects methodological issues (such as remote delivery of ABM; Heeren et al., 2015). Perhaps self-related processing is more relevant to SAD than more general attentional processes. Indeed, a range of alterations in self-processing in SAD deserve better characterization and further exploration (Stein, 2009; Gaebler et al., 2014).

## Neurocircuitry of Sad

Multiple brain imaging studies of SAD have contributed to our understanding of the neurocircuitry of SAD (Stein and Stein, 2008). A recent meta-analysis of functional magnetic resonance imaging (fMRI) studies found that most significant areas of activation during emotional vs. neutral stimuli in individuals with SAD compared to controls included bilateral amygdala, anterior cingulate, and globus pallidus (Hattingh et al., 2012). These findings are consistent with an animal literature emphasizing the involvement of the amygdala in fear conditioning (Davis, 1990). There are, however, also some imaging findings in individual studies of SAD, such as increased activation in temporoparietal regions, including posterior superior temporal sulcus (pSTS) and supramarginal gyrus (SMG)/temporo-parietal junction (TPJ), which are consistent with increased bodily self-consciousness (Gaebler et al., 2014).

In addition, there is evidence that during fMRI, individuals with SAD demonstrate increased blood-oxygenation level-dependent (BOLD) responses relative to controls, in medial prefrontal cortex (mPFC) and amygdala after self-referential criticism, but not after self-referential praise, or after other-referential criticism or praise (Blair et al., 2008). In a study of individuals with subclinical social anxiety, self-referential processing was associated with activity in the mPFC (thought to be involved in self-representation), posterior cingulate and temporal poles (Abraham et al., 2013). Furthermore, compared to low socially anxious individuals, highly socially anxious individuals demonstrated increased activation of mPFC, TPJ, and temporal pole when they focused their attention inwardly rather than outwardly during a simulated social situation (Boehme et al., 2015). Finally, increased amygdala activity in SAD is reduced after group CBT (Furmark et al., 2002) and after internet-CBT (Mansson et al., 2013).

The insula appears to play an important role in interoceptive processing and self-consciousness (Ronchi et al., 2015). It is notable therefore that a number of functional imaging studies have found altered activity in the insula in SAD, and that after pharmacotherapy with either a serotonin selective serotonin reuptake inhibitor (SSRI) or a reversible inhibitor of monoamine oxidase inhibitor (RIMA), SAD patients had decreased insula activation at rest (Warwick et al., 2006). Other SAD studies have found that activation of various structures including insula and temporo-parietal regions was normalized by CBT (Klumpp et al., 2013) as well as by emotional regulation techniques such as self-focused reappraisal (Brühl et al., 2013; Gaebler et al., 2014). Given the growing literature on the neurocircuitry of self-related processes and self-focused attention (Stein, 2007; Northoff et al., 2011), additional work is needed to link alterations in structural and functional brain imaging in SAD with assessments of self-consciousness and related processes.

## Neurochemistry of Sad

Multiple neurotransmitter and neuroendocrine systems have been investigated in SAD (Fox and Kalin, 2014). There is evidence that SAD is characterized by alterations in both the dopaminergic and serotonergic systems in particular (Stein et al., 2002; Frick et al., 2015). Neuropeptides such as oxytocin may well be important in mediating social anxiety. Still alterations in such systems have not yet been shown to be specific for SAD, and while SAD has moderate heritability, the particular neurogenetic variations underlying this condition also remain uncertain (Fox and Kalin, 2014).

Broadly speaking, neurochemical systems clearly play an important role in brain regions that mediate both SAD as well as self-related processes. Furthermore, it is possible that neurotransmitter systems involved in SAD, such as the dopaminergic and serotonergic systems, also have a role in self-related processes such as corporeal awareness (Albrecht et al., 2011). That said, few studies have specifically integrated measures of neurochemical systems in SAD together with assessment of alterations in self-consciousness, self-focused attention, or related phenomena in SAD.

Blushing and gaze aversion may be particularly important symptom of SAD, and they may also be closely related to self-consciousness (Stein and Bouwer, 1997a). There is some preliminary work on the neurochemistry of blushing, although a good deal more investigation is needed to fully understanding the psychobiology of blushing, and possible psychobiological abnormalities in patients with persistent blushing in the context of SAD (Stein and Bouwer, 1997a). For now, given the absence of studies that directly address this issue, it again seems speculative to draw associations between neurotransmitter and neuroendocrine alterations in SAD, and specific SAD symptoms (such as blushing and gaze version) that are associated with increased self-consciousness or self-focused attention.

## Evolutionary Psychiatry and Sad

Any question in biology may be addressed by considering both the relevant proximal or physiological explanations (e.g., the psychobiology of SAD) as well as the related distal or evolutionary explanations (e.g., the adaptive value of social anxiety; Mayr, 1982). A growing literature has explored the adaptive value of anxiety, and the implications of an evolutionary perspective for understanding anxiety and related disorders such as SAD (Stein and Bouwer, 1997b; Stein and Nesse, 2015). From this perspective, social anxiety is adaptive insofar as it appropriately alerts humans to social threat, and ensures successful negotiation of social hierarchies. It is relevant to consider here whether the evolutionary literature on social anxiety bears any relationship to that on self-consciousness.

It is notable that blushing was comprehensively discussed by Darwin (1872). From an evolutionary perspective, blushing can be considered an “appeasement display” which has adaptive value in decreasing aggression between conspecifics (Stein and Vythilingum, 2007). SAD may be characterized by a low threshold for such displays. It is significant, perhaps, that both blushing and social anxiety increase during adolescence, a time when self-related processing becomes more sophisticated and when public self-consciousness often increases. Such changes may speculatively be mediated by alterations in mPFC and striatum-mPFC connectivity during adolescence (Somerville et al., 2013). While human neurodevelopment can be understood from an adaptive life-course perspective, this period in life certainly represents a challenge for emotional self-regulation (Stein, 2008).

## Conclusion

Social anxiety and SAD appear to be phenomena that are highly relevant to a consideration of the psychobiology of self-consciousness. Given how important social anxiety is in everyday life, and how prevalent and disabling SAD is (Stein et al., 2010), much additional work is needed to fully delineate their psychobiology. Nevertheless, the literature on SAD has begun to address the psychobiology of alterations in self-related processing and self-focused attention, and it is remarkable that a number of investigations of this disorder have highlighted altered activity in mPFC (involved in representation of the self), insula (involved in interoceptive processing) and in other structures that play a role in bodily self-consciousness, as well as the potential value of interventions such as SSRIs and self-focused reappraisal in normalizing such changes. It may be valuable for future investigations to more closely address associations between psychobiological alterations and changes in self-related processing in SAD, in order to shed additional light on both SAD and self-consciousness.

## Conflict of Interest Statement

Dr. Stein has received research grants and/or consultancy honoraria from AMBRF, Biocodex, Cipla, Lundbeck, National Responsible Gambling Foundation, Novartis, Servier, and Sun.
